# Purification of Ionic Liquid [mTBDH][OAc] Utilizing the Short-Path Distillation Technique

**DOI:** 10.1021/acsomega.5c04442

**Published:** 2025-09-09

**Authors:** Mohammed Saad, Inge Schlapp-Hackl, Petri Uusi-Kyyny, Ville Alopaeus

**Affiliations:** † School of Chemical Engineering, Department of Chemical and Metallurgical Engineering, 174277 Aalto University, P.O. Box 11000, Aalto FI-00076, Finland; ‡ School of Chemical Engineering, Department of Bioproducts and Biosystems, 174277 Aalto University, P.O. Box 11000, Aalto FI-00076, Finland

## Abstract

The Ioncell process is an innovative approach to producing sustainable textiles using an ionic liquid (IL) as a solvent, but this process generates side products and impurities that have negative effects on its solvent dissolution capability. Therefore, the purification of the IL is of utmost importance. 7-Methyl-1,5,7-triazabicyclo[4.4.0]­dec-5-enium acetate, [mTBDH]­[OAc], is a suitable alternative to replace existing solvents for the application of cellulose dissolution in the Ioncell process. 7-Methyl-1,5,7-triazabicyclo(4.4.0)­dec-5-ene (mTBD) is an expensive base, and consequently, a closed loop operation, without any losses, is desirable. Additionally, high recovery of the base and effective removal of the impurities from the IL are necessary for making the process sustainable. Understanding the interaction of the IL with impurities is essential to purify mTBD and, thereby, the IL. This study focuses on the purification of [mTBDH]­[OAc] in the presence of different impurities. KCl, NaCl, CaCl_2_, lactic acid, xylan, and the hydrolysis products, 1-[3-(methylammonio)­propyl]-1,3-diazinan-2-onemium acetate (H-mTBD-1) and 1-(3-ammoniopropyl)-3-methyl-1,3-diazinan-2-onemium acetate (H-mTBD-2), were the impurities utilized to understand the IL-impurity interactions. Suitable conditions for purification were determined using a short path distillation (SPD) unit while varying the feed flow rate to investigate the recovery of the base mTBD at different mass fractions. SPD results indicated that the highest recovery fractions (0.97–1.00) were observed at the lowest flow rates­(0.378 kg/h). Experimental results also confirmed that approximately 75–100% of the base mTBD can be recovered in most cases. Overall, the results of the experiments confirm that the impurities K, Na, Ca, xylan, and lactic acid can be removed from the feed using an SPD unit.

## Graphical abstract

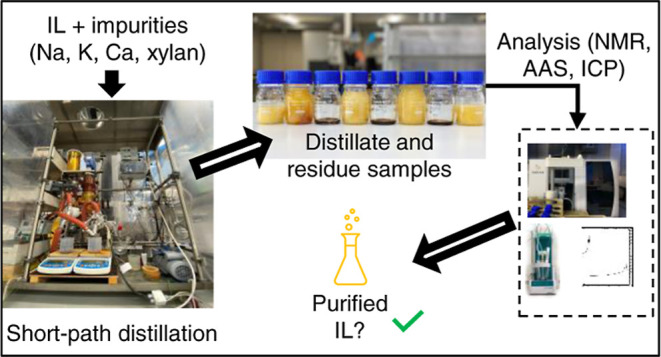

## 1 Introduction

The world population growth will result in the increased consumption of natural and man-made resources. To reduce fiber footprints across the globe, sustainability and circular economy-based research and initiatives need to be promoted. As the demand for renewable and biodegradable materials is increasing, so is the need for efficient and ecological solvents for the processing of lignocellulose.^1^ Cellulose-based fibers have desirable properties such as recyclability, sustainable sourcing, wearing comfort, remarkable absorption/desorption properties, etc.^2^

The processes that are currently dominant in the man-made cellulose fiber market are the viscose and the lyocell process.^3^ Up to now, the lyocell process has been the only commercial alternative to the environmentally harmful viscose technique. The produced lyocell fibers show a high degree of orientation, resulting from strong stretching of the liquid filament at the spinning step. Compared to viscose fibers, lyocell fibers show better mechanical properties.^3^ Sixta et al. introduced a process to produce cellulose regenerated fibers via the use of the well-known lyocell type dry-jet-wet spinning technique, whereby they are manufactured from an ionic liquid (IL)–cellulose solution.^2,4,5^

Due to the low processing temperature, a much lower energy consumption is needed to produce Ioncell fibers compared to the Lyocell process. Also, the excellent dissolution behavior of the cellulose polymers in the IL helps to reach higher cellulose concentrations and further to reduce the needed amounts of chemicals compared to the Lyocell technique.^6^ Additionally, the Ioncell fibers show remarkable mechanical properties, which offer the possibility to enter the market for different applications.^2,5^ In short, the Ioncell process showed excellent results and could help address the growing demand of increasing consumption of global resources.^6^ Most works promote the utilization of ILs as alternative cellulose solvents.^7^ This observation holds true without considering the effect of the impurities or side products that would result from a continuous process. These impurities would accumulate in the IL, negatively affecting the overall process.^8–11^

During the pulp extraction of the Ioncell process, different inorganics found in wood can be removed, and traces are currently detected after the purification step.^12^ In the Ioncell process, the IL is removed and collected after the cellulosic fiber regeneration. Next, the sample is fractionated and recycled. However, some of the cations, which come from the pulp, will also seep into the IL and accumulate during the recycling process.^13^

The selection and success of a new solvent for the lyocell process certainly depend on its recyclability.^14^ Irrespective of the potential to dissolve cellulose, high recovery rates need to be attained to take advantage of ILs in processes for industrial adaptation. 1,5-diazabicyclo[4.3.0]­non-5-enium-acetate [DBNH]­[OAc], 7-methyl-1,5,7-triazabicyclo[4.4.0]­dec-5-enium acetate [mTBDH]­[OAc], and 1,8-diazabicyclo[5.4.0]­undec-7-enium acetate [DBUH]­[OAc] have been identified as excellent solvents for the lyocell type Ioncell process.^14–17^ Elsayed et al. studied the recyclability and the change in the dissolution capacity of [mTBDH]­[OAc] compared with [DBNH]­[OAc] within five spinning cycles. Thereby, the IL solutions were recovered by thermal treatments under reduced pressure. [mTBDH]­[OAc] revealed excellent results. Compared to [DBNH]­[OAc], it showed significantly higher thermal and hydrolytic stability. The recovery cycles did not impair the dissolution ability.^18^ Whereas the thermal recovery of [DBNH]­[OAc] revealed significant hydrolysis products that killed the solvation power of the IL due to increased volatility and thereby, an increased readiness to form the azeotrope mixture and therefore, losing the ability to dissolve cellulose.^19^

Inorganic anions (e.g., chlorides, phosphates, and sulfates) and cations (e.g., sodium, potassium, and calcium) from the pulping process could be released in the coagulation bath during spinning and even concentrated during solvent recovery leading to salting-in/out phenomena of the system.^20–22^ The phosphates and sulfates were not considered in the current study as the IL that is utilized is acetate-based and not sulfate- or phosphate-based. Hydrolysis products, 1-[3-(methylammonio)­propyl]-1,3-diazinan-2-onemium acetate, [H-mTBD-1], and 1-(3-ammoniopropyl)-3-methyl-1,3-diazinan-2-onemium acetate, [H-mTBD-2], resulting from side reactions at elevated temperatures and the precursor of mTBD, 1,5,7-triazabicyclo [4.4.0] dec-5-ene (TBD), which is a residue from the synthesis process of the superbase mTBD, are also considered as impurities.^23^ It is undesirable to have an acid-rich solvent for cellulose dissolution, as acetic acid is not a cellulose solvent. The ability of the acid to establish hydrogen bonds with the ion pair in the liquid solution leads to an acid-rich solvent.^24^ Additionally, residual water from the recycling step can also affect the dissolution process. Additional impurities such as textile dyes,^25^ finishing agents, and pulping chemicals^26^ could be present and influence the performance and properties of the recycled fibers but these are out of the scope of the current study.

In our work, short path distillation (SPD) is used for recovering the IL in the presence of the above-mentioned impurities. SPD is a distillation technique suitable for the separation and purification of thermally unstable liquids and liquids having low vapor pressure and high molar mass without the hazard of thermal decomposition.^27^ The short residence time of the liquid in the evaporator takes place through the distribution of the liquid in the form of a thin wiped film.^28^ The short distance between the evaporator and the condenser surfaces alongside vacuum in the evaporator results in a negligible thermal decomposition, and the distillation occurs at rates that can be feasibly applied with existing technology. ILs have been previously recovered using SPD units as provided in Table 1.

**Table 1:** Applications of Distillation in IL Recovery

application	SPD unit	operation parameters	results	refs
distillation of EEIM OAc	patented molecular distillation	*p*: 0.05mbar	distillate 190g	^31^
		*T*_distillation_:170 °C	purity >95%	
		feed rate 200 g (2 h)	residue 10g	
distillation of EMIM OAc	patented molecular distillation	*p*: 0.05mbar	distillate 170g	^31^
		*T*_distillation_: 170 °C	purity> 95%	
		feed rate 200 g (4 h)	residue 30g	
distillation of TMGH CO_2_Et	Büchi Kugelrohr SPD	*p*: 1.3mbar	distillate 99.4%	^32^
		*T*_distillation_:100–200 °C	purity >99%	
		feed 3 g (1 h)	residue 0.6%	
distillation of DBNH CO_2_Et	A Kugelrohr apparatus	*p*: 1mbar		^12^
		*T*_distillation_: 170 °C		
		feed: 1.5 g (acid/base = 1:1)		
distillation of Bmim OAc, EeimOAc	An SPD apparatus	*p*: 0.05mbar	distillate: 90%	^33^
distillation of EmimOAc	A laboratory Kugelrohr	*T*_distillation_: 170 °C *p*: 0.3mbar	residue: 10%	
		*T*_distillation_: 180 °C	distillate: 100%	
recovery of AmimCl in homogeneous cellulose acetylation	pope 2″ diameter laboratory wiped-film short-path molecular still	*p*: 0.13mbar	purity: 99.5%	^34^
		*T*_distillation_: 95 °C		
		*T*_feed_: 80 °C		
		scrapper speed: 440 rpm		
		feed rate: 1 mL/min		

This paper focuses on the removal of different impurities from [mTBDH]­[OAc] and determines how the impurities mentioned above affect the purification of the IL. This is done by adding the above impurities to pronounce their effect in the experiments. One of the objectives of this study is also to investigate if it is possible to purify the IL [mTBDH]­[OAc] in the presence of these impurities with minimal loss via SPD. The study also aims to determine the parameters for carrying out the SPD experiments and the temperature at which thermal degradation of the IL (A-mTBD formation) takes place in the system. Previous studies have indicated that degradation of the superbases occurs as a result of hydrolysis, which in turn affects cellulose dissolution if the hydrolysis product content gets too high.^8,29,30^ Further analyses are carried out to identify how the different components are distributed in different streams and how the feed mixture changes with respect to the different impurities.

## 2 Materials and Apparatus

### 2.1 Chemicals and Reagents

Equimolar [mTBDH]­[OAc] (7-methyl-1,5,7-triazabicyclo[4.4.0]­dec-5-enium acetate) was prepared at Aalto University by adding glacial acetic acid (100% purity, Merck, Germany, 60.05 g/mol; CAS: 64-19-7) to mTBD (153.22 g/mol; CAS:84030-20-6; 7-methyl-1,5,7-triazabicyclo[4.4.0]­dec-5-ene) (BOC Sciences, USA) in a controlled temperature reaction.^14^ The mTBD hydrolysis products (H-mTBD-1) and (H-mTBD-2), which accounted for 2% of the feed in the experimental runs, were prepared in-house with a molar composition of 74% H-mTBD-1, 19% H-mTBD-2, and 7% mTBD. Sodium chloride (58.44 g/mol; CAS:7647-14-5; NaCl) was obtained from VWR Chemicals with a purity of 99.5%. Potassium chloride (74.56 g/mol; CAS:7447-40-7; KCl) was supplied by Oy FF-Chemicals Ab. Calcium chloride (110.98 g/mol; CAS:10043-52-4; CaCl_2_) was purchased from Sigma-Aldrich with a purity of 97%. Additionally, lactic acid (90.08 g/mol; CAS:50-21-5; C3H6O3; 92% purity) used in the experiments was provided by Honeywell Fluka. Also, xylan from birchwood (150.13 g/mol; CAS:9014-63-5; C_5_H_10_O_5_) used in the experiments was purchased from Sigma-Aldrich and contained xylose residues ≥90% analyzed by High-Performance Anion-Exchange (HPAE) chromatography. Finally, the water used for the experiment went through a purification system in-house, including softening, demineralization, reverse osmosis, and UV disinfection. The tested water samples indicated conductivity in the range of 1.6–1.7 μS/cm and TOC <0.5 mg/L.

The salts KCl, NaCl, and CaCl_2_ were gravimetrically analyzed for the water content within them. The masses of the salts were measured initially and then placed in a GC oven overnight at 200 °C. The salt samples were weighed the next day to determine the dry mass. The weighed samples indicated that CaCl_2_ had 6.65% water, while KCl and NaCl had 0.05% water present in them.

**Figure 1:**
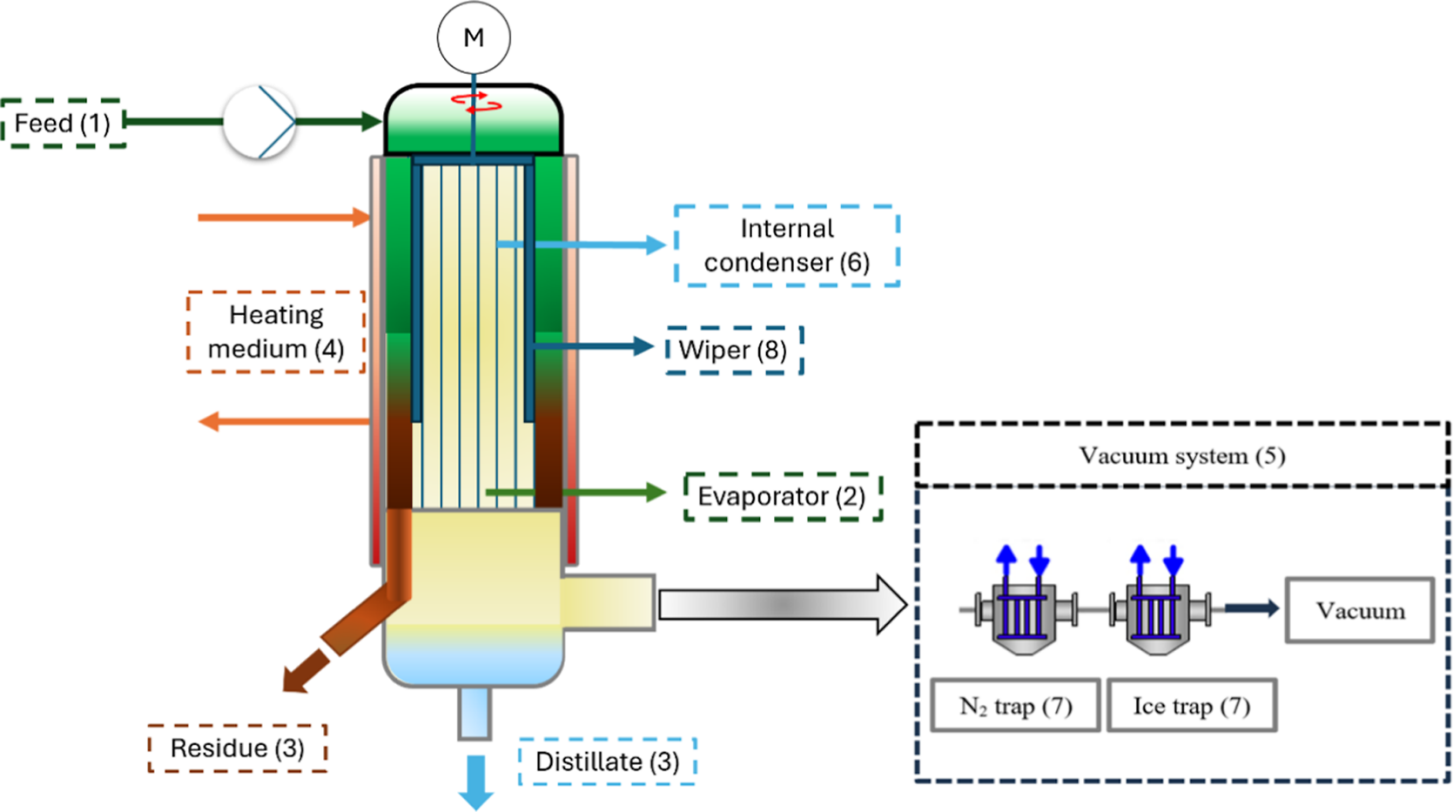
Components of the modified KDL 5 laboratory plant used in experiments.

### 2.2 Short Path Distillation

The SPD setup used in the experiment is a modification of the UIC_KDL5 made by UIC GmbH with an additional cold trap to increase the separation efficiency.^35^ Figure 1 shows the main components of the experimental SPD unit. The SPD setup consists of a feed pump with a dosing vessel (1), a heatable distillation part (2), distillate and residue systems (3), heating units for the evaporator, feed system, residue, and distillate (4), and a rotary vane vacuum pump set (5).

**Figure 2:**
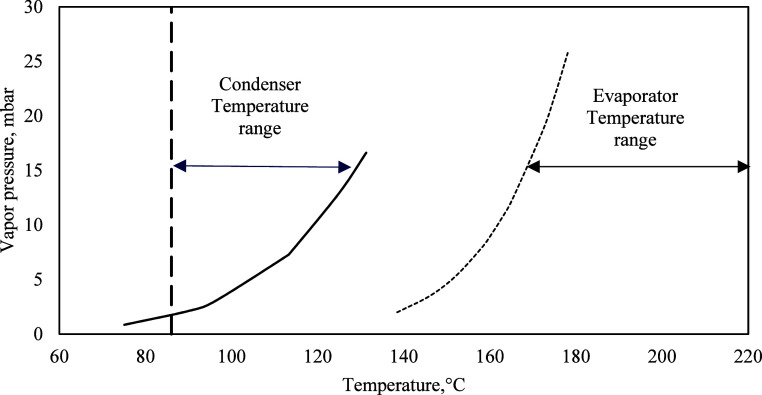
Operating temperature range selection for condenser and evaporator. Legend: mTBD vapor pressure (-), melting point of IL (- -), mTBD complex vapor pressure (--). Data from Baird, Z. S.; Uusi-Kyyny, P.; Witos, J.; Rantamäki, A. H.; Sixta, H.; Wiedmer, S. K.; Alopaeus, V. J. Chem. Eng. Data 2020, 65, 5, 2405–2421. Copyright © 2020 American Chemical Society.

The feed system comprises a 2L feed vessel that can be heated up to a maximum of 150 °C, a circulation pump, a feed heating device, and a circulation thermostat.

The SPD unit consists of a glass apparatus with a double jacket for heating the evaporator surface as well as the outlet nozzles for the distillate and residue with heat transfer oil. The surface area of the evaporator is 4.8 dm^2^ and the surface area of the condenser (6) is 6.5 dm^2^ with a temperature range of 25–350 °C. The condenser also consists of a circulation pump and a circulation thermostat with a heating oil bath for the evaporator jacket.

The flanged cold trap (7) for ice is connected to a round-bottomed glass flask for the collection of condensates. The roller wiper system (8), having a drive motor with a manually adjustable speed (200–500 rpm), also forms part of the setup.

The SPD experiments were carried out at different flow rates as the feed pump could be adjusted between 0 L/h and 1.8 L/h. The feed temperature was set at 95 °C throughout all of the experimental runs. Varying the flow rates of the pump also resulted in a slight variation of the pressure inside the system. The distillation unit consists of a glass apparatus with a double jacket for heating the evaporator surface as well as the outlet nozzles for the distillate and residue with heat transfer oil. The roller wiper system of the distillation had a drive motor with a manually adjustable speed. The pumps and pipes had insulation around them to prevent heat loss. Additionally, two electronic control units (PID, Type-K, 16A, Meyer Vastus Oy, Finland) with thermal tapes (1.0 m, 450 °C, Horst GmbH, Germany) were connected to the distillate and residue outlets to control the outlet temperatures. The control units were set at 100 °C to prevent crystallization at the outlets of the SPD unit. The temperature of the condenser and residue units was set at 100 °C each. Furthermore, the flanged cold trap for ice and nitrogen is connected to a round-bottom glass flask for the collection of condensates.

The evaporator temperature was kept at 200 °C based on the vapor pressure curve shown in Figure 2. The vapor pressure curve is based on the vapor pressure data provided by Baird et al.^37^ In our earlier work, during batch distillation of this IL, pure base mTBD is initially obtained in the distillates. As the process continues, the residue starts accumulating as a complex with an acid/base ratio of 3:2. According to this work, the minimum evaporator temperature required to evaporate the complex (acid: base −3:2) is 160 °C.^16^ In Phuong’s work, the operating pressure was slightly lower in the range of 1–2 mbar, which, alongside the combination of the selected temperatures, feed flow rates, and heat transfer, could have resulted in incomplete evaporation of the complex.^16^ Based on Figure 2, the condenser temperature is determined by the vapor pressure of mTBD and the melting point of the IL. The temperature needs to be high enough (>90 °C) to prevent crystallization of the IL. The temperature also needs to be low enough to condense the mTBD. The distillation temperature is determined by the boiling point of the lowest vapor pressure component, which is the complex. The upper limit of the distillation temperature is determined by the decomposition temperature of the IL, where it decomposes to A-mTBD. According to Figure 2, there will be a separation of the mTBD from the complex in the temperature range between the condenser and evaporator temperatures.

The operating pressure of the experiments was kept in the range of 9–10 mbar. Also, the selection of the operating pressure was based on the vapor pressure of pure mTBD, the complex, and the condenser temperature. If the pressure is too low, excessive material losses to the traps would occur. The SPD unit was heated up to the desired experimental conditions. This also ensured that no moisture content would be detected in the device. After the desired temperatures were attained, the valves of the cold traps to the atmosphere were closed to reach the selected pressure. The vacuum pump was turned on to evacuate the unit until the system stabilizes at the desired pressure. Once the required temperatures and pressures are attained, the ice trap and nitrogen trap are filled with ice and nitrogen, respectively. The feed was then poured into the dosing vessel, and the feed pump was turned on. To achieve short hold up times and high evaporation rates, the wiper rate needs to be controlled so that there is an optimal film distribution on the evaporator surface. The wiper rate was fixed at 300 rpm in all the experimental runs. Lower wiper speed (<200 rpm) would result in poor film distribution, leading to uneven evaporation, while higher speeds (>400 rpm) could result in excessive splashing, leading to inefficient separation. Therefore, selecting a wiper speed of 300 rpm balances evaporation efficiency and uniformity of the thin film, thereby maximizing mass transfer. Also, 300 rpm ensures short residence time, which is critical for heat-sensitive compounds like cellulose derivatives.^38^ The feed vaporizes in the evaporator and flows to the condenser, where the light components are collected as a distillate. The heavier components that do not vaporize are removed in the bottom product as a residue. The condenser is unable to condense the water under these conditions, which as a result gets transferred to the traps. All of the samples from the feed to the products and traps were weighed and analyzed depending on the impurities present in them.

The density of the IL [mTBDH]­[OAc] was selected for the mass balance calculations as it was the major component of the feed by using the liquid density values from the work of Baird et al.^39^ The data were selected in the range where the molar acid to base ratio is 49–51, which is close to the acid–base distribution used in the feed. Interpolating the data, the density was determined for the evaporator temperature in use, 473.15 K, which was taken from Baird et al.’s work.^39^

### 2.3 Analysis of Experimental Samples

#### 2.3.1 NMR Analysis

All of the samples were analyzed using ^1^H NMR. The NMR Spectrometer used for the analysis is a Bruker AV III 400. A small amount (1–5 mg) of sample is diluted using dimethyl sulfoxide (DMSO–d_6_) in a precision NMR glass tube. The sample is mixed to ensure that the sample remains homogeneous. The MestReNovax64 software was used for analyzing the NMR results. The analyzed samples are provided in the Supporting Information from Tables S1–S8.

#### 2.3.2 AAS Analysis

The samples containing sodium and potassium in the experiments were quantified using an AAS (Varian AA240). Depending on the elements, different standards were produced to obtain a satisfactory linear calibration (Sodium Standard for ICP: 1000 mg/L Na in nitric acid; Potassium Standard for ICP: 1000 mg/L K in nitric acid obtained from Merck). The following concentrations of the standards were produced: 0.1, 0.3, 0.6, 0.9, 1.2, and 1.5 mg/L. Initially, the solidified samples collected after SPD had to be first melted in an oven at 80 °C. 1 g portion of each sample was then transferred to a 50 mL volumetric flask and diluted with deionized water to reach 1 g/50 mL. Depending on the concentration of the mixture, the samples were diluted accordingly to reach the range of the standards. The potassium is measured at 766.5 nm, and the sodium is measured at 589.0 nm. The sample was injected three times, and the average value was calculated.

#### 2.3.3 ICP Analysis

The samples containing calcium were analyzed using an ICP PerkinElmer, Optima 7100DV. The setup allows for multielement measurement. For calcium calibration, 1000 mg/L Ca in nitric acid purchased from Merck was used, and the following concentrations were used: 0.5, 1, 5, 10, 15, and 20 mg/L. A similar procedure is repeated with ICP sample preparation as with AAS. The used wavelength for Ca was 317.933 nm. Calibration and calculation are carried out similarly to AAS.

#### 2.3.4 High-Performance Anion-Exchange Chromatography with Pulsed Amperometric Detection Analysis

The carbohydrates and lignin amounts were determined via a well-known analytical procedure of the National Renewable Energy Laboratory.^40^ This method is used for the analysis of the hemicellulose xylan by converting it to xylose. A two-step acid hydrolysis is utilized to convert the xylan to xylose, so that it can be quantified easily. The samples from the experiment are in a crystalline form. Initially, they are melted in an oven at 85 °C. This is followed by dilution of the samples using a dilution factor of 7.3. In HPAEC-PAD, sugars are measured using a standard curve that works optimally when the sugar levels are in the range of 1 mg/L–50 mg/L. If the sample has more sugar than that, it needs to be diluted to fall within this range. Next, 3 mL of sulfuric acid (72% w/w, commercially available as a reagent for the determination of fluorine from Fluka #00647) is added to the hydrolyzed sample. 84 mL of distilled water is added and the samples are autoclaved by the use of a Systec DE 23 Autoclave at 120 ± 3 °C for an hour. The samples are then left to cool at room temperature. Next, the samples are diluted and filtered 1:100. The samples are filtered with 0.2 μm filters and measured with a Biorad Aminex HPX-87H column.

In general, HPAEC-PAD is used only for analyzing acidic/water-based samples. Consequently, to confirm the method, an initial analysis was carried out using a synthetically prepared mixture of the IL sample mimicking the impurity profile of the experimentally utilized IL. The sample was analyzed by the Dionex ICS-3000 HPAEC-PAD device, and the analysis confirmed that it is possible to use this technique for analyzing IL-based samples.

#### 2.3.5 Karl Fischer Titration

The water content of the samples was measured by KF titration with an OMNIS titrator. Table 2 shows the water content in the different feed mixtures, which were measured in the remaining samples at the end of the final experiment.

**Table 2:** Water Content in Different Experimental Feeds

experiment	water content (wt %)
3	0.12
4	0.89
6	0.67
7	1.00
8	3.395

### 2.4 Theoretical Calculations

The recovery fractions from the measured samples were calculated using eq 1 as follows

1 $$Recovery\;fraction=\frac{Mass\;of\;distillate\left(g\right)}{Mass\;of\;distillate\left(g\right)+Mass\;of\;residue\left(g\right)}$$

The split fractions of the salts in the distillate to that of the salts in the residue were calculated using eq 2 below

2 $$Split\;fraction\;of\;A\;\left(K,Na,Ca\right)=\frac{Mass\;fraction\;of\;A\;in\;distillate}{Mass\;fraction\;of\;A\;in\;residue}$$

Additionally, the results of the NMR analysis had to be converted to mass % using eq 3 as follows

3 $$Mass\%=Molar\;mass\;of\;A^{*}mol\%$$

The mass fraction of each component was then obtained by using eq 4

4 $$Mass\;fraction\;of\;A=\frac{m_{A}}{m_{total}}$$

where *m*_A_ is the mass of component A in g.

The amount of salts in the mass balance calculation is carried out using eq 5

5 $$Mass\;of\;element\;\left(K,Na,Ca\right)={\frac{Atomic\;mass}{{\left(Molar\;mass\right)}_{salt}}}^{*}m_{salt}$$

where *m*_salt_ is the mass of the salt in g.

The yield of mTBDH in the distillate is calculated using eq 6

6 $$Yield_{D}=\frac{Mass\;fraction\;of\;mTBDH\;in\;distillate^{*}mass\;of\;distillate}{Mass\;fraction\;of\;mTBDH\;in\;feed^{*}mass\;of\;feed}$$

## 3 Discussion and Results

The recovery of [mTBDH]­[OAc] in the presence of KCl, NaCl, CaCl_2_, lactic acid, and xylan and the corresponding hydrolysis products, H-mTBD-1, and H-mTBD-2 was investigated using the short-path distillation unit. In total, eight sets of experiments were run at three different flow rates, except experiment 2, where four different flow rates were tested. In sum, 25 runs were carried out while keeping the parameters like feed temperature, evaporation temperature, operating pressure, wiper speed, and condenser temperatures constant. The matrix of the experimental runs is provided in Table 3.

**Table 3:** Feed Component Measurements in Different Experimental Runs at *T*_evaporator_ = 200 °C and *p* = 9.14 mbar

experiment #								
components	1	2	3	4	5	6	7	8^a^
measured amount (mass %)								
[mTBDH][OAc]	98.1	98.16	97.02	96.88	98.3	97.32	96.12	93.62
H-mTBD-1, H-mTBD-2	1.9	1.62	2	2.98	1.62	2.10	1.90	2.18
NaCl		0.11				0.10	0.29	
KCl		0.11				0.11	0.31	
CaCl_2_			0.98			0.10	0.29	0.50
xylan				0.14		0.15	0.29	
lactic acid					0.08	0.12	0.80	0.60
water								3.10

a *T*_evaporator_ = 220 °C.

Seven experiments were carried out at an average pressure of 9.14 mbar and an evaporation temperature of 200 °C. In every experimental run, the lowest flow rate provided the highest recovery of mTBD. The recovery fractions from all the experimental runs are combined in Figure 3, which shows the gradual decrease in recovery as the flow rate increases. The SPD results in SI Table S9 showed that it is possible to separate K, Na, Ca, lactic acid, and xylan from the IL using this method.

**Figure 3:**
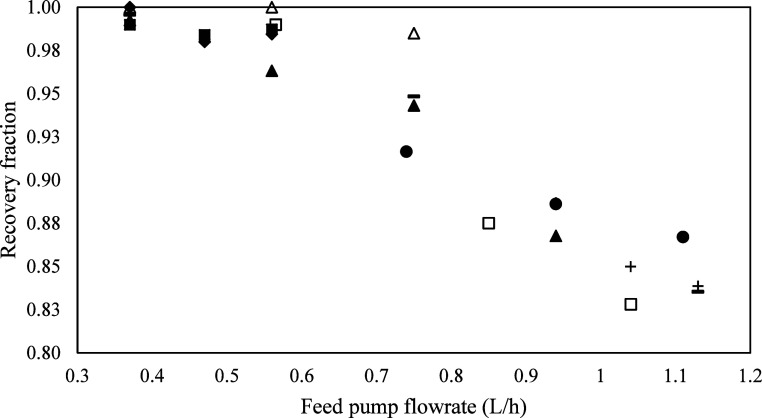
Recovery fraction vs flow rate for all experiments. Experiments: 1 (●), 2 (▲), 3 (-), 4 (△), 5 (■), 6(⧫), 7(□), 8­(+).

The yield of mTBD is plotted against the residence time in Figure 4, indicating that an increase in residence time increases the yield/recovery of the base. Overall, the mTBD distillate yield is in the range of 51.6%–104.4% depending on the feed flow rate. This happens in most cases, confirming that recovery of the base mTBD is possible. The above results are limited to the current setup’s capacity. Different equipment with higher capacity could improve the recovery fractions at higher flow rates. Introducing a second SPD unit in series would be beneficial to minimize the losses and increase the yield of mTBD in the distillate.

**Figure 4:**
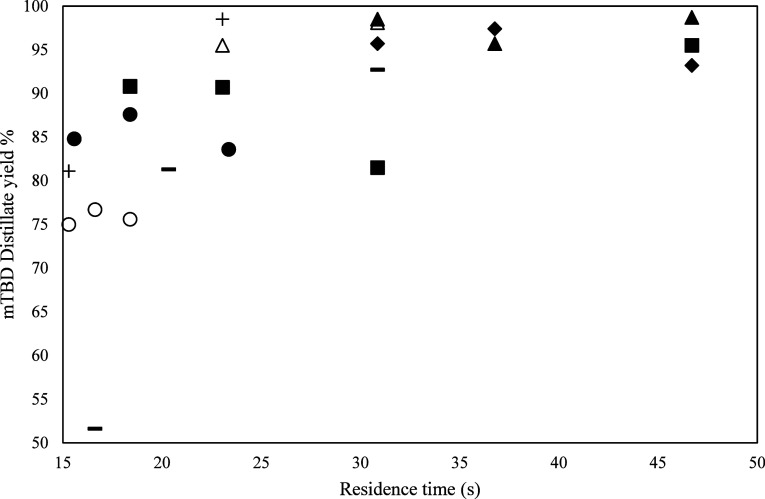
mTBD distillate yield vs residence time for all experiments. Experiments: 1 (●), 2 (■), 3 (+), 4 (△), 5 (▲), 6(⧫), 7­(-), 8(○).

The formation of mTBD over 100% in the distillate in some cases could be due to the reverse reactions of the decomposition products H-mTBD-1 or H-mTBD-2 or both, as indicated in Figure 5, which is the hydrolysis pathway of [mTBDH]­[OAc].^18^ The experimental results indicated that some of the results did not conform to the established pathway. Recent studies showed the stabilization of the bases DBN and mTBD by an azeotrope formation in mixtures with acid–base ratios of 5:3 and 3:2, respectively. The IL [mTBDH]­[OAc] demonstrated substantially better resistance to hydrolysis reactions, and alterations carried out with this recovered IL indicated good cellulose dissolution (>97%).^41^ The target was to have an mTBD distillate yield as high as possible, and this is possible when the feed flow rates are low. The estimated residence time in the evaporator at 220 °C is 15s if the assumed thickness of the thin film is 0.1 mm at a feed flow rate of 1.13 L/h. Assuming a thickness of 0.1 mm for the thin film of the evaporator and the surface area of 4.8 dm^2^, the residence times were calculated for all the experiments.

**Figure 5:**
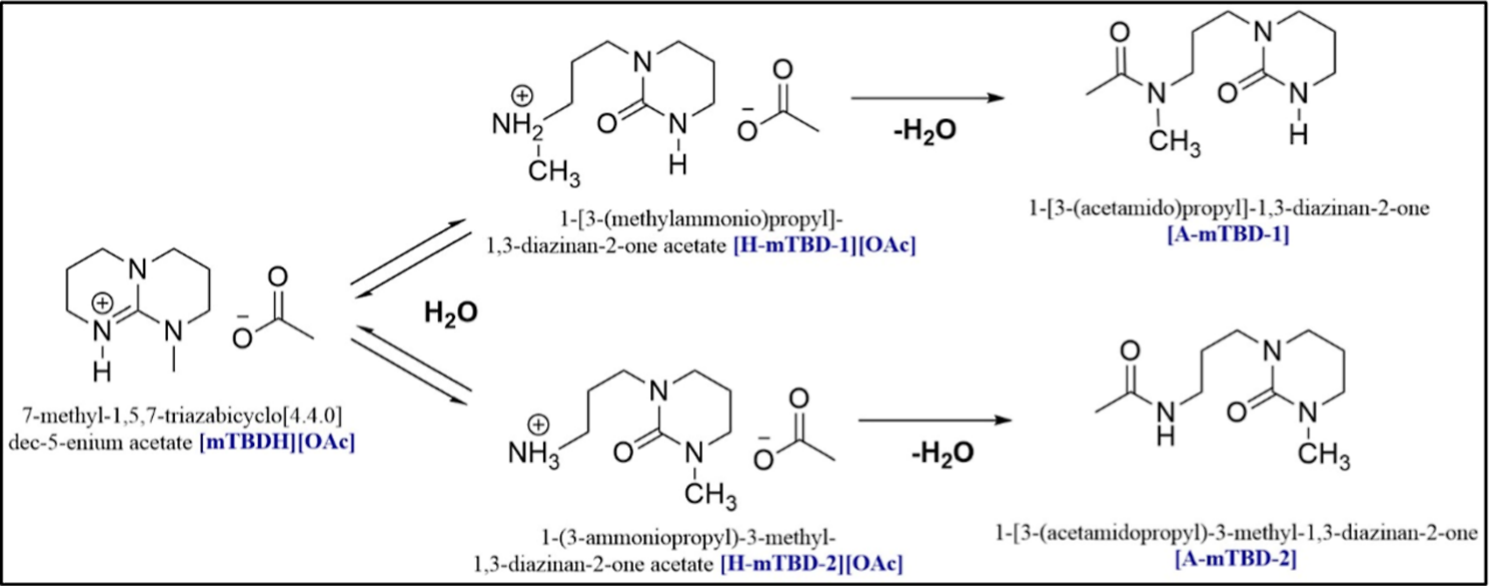
Hydrolysis pathway of [mTBDH]­[OAc].^18^ Reprinted with permission from Elsayed, S.; Hellsten, S.; Guizani, C.; Witos, J.; Rissanen, M.; Rantamäki, A. H.; Varis, P.; Wiedmer, S. K.; Sixta, H. ACS Sustainable Chem. Eng. 2020, 8, 37, 14217–14227. Copyright © 2020 American Chemical Society.

### 3.1 Experiment 1

Experiment 1 was carried out at a *p* = 9.14 mbar with the feed and evaporator temperatures set at 95 and 200 °C, respectively. The amount of residue (11.71, 16.05, and 41.55 g) increases with increasing flow rate (0.766, 0.961, and 1.113 kg/h), pointing to the fact that a lower flow rate improves the recovery of the IL. The high recovery at low flow rates is because at low flow rates, the wall film formation is thinner and more uniform, which is more favorable for the separation of light and heavy fractions. According to the NMR analysis (Supporting Information) and mass balance of the collected samples, it was observed that over 80% of the feed in all 3 cases ended up in the distillate. The major component in the liquid nitrogen trap was water (99.9 mol %), even though there were traces of mTBD and HOAc. The peaks of mTBD and HOAc were small in comparison to the amount of water. On the other hand, the ice trap contained approximately 55.8 mol % of mTBD and 40.2 mol % of HOAc. Also, in the operation of this experiment, no transformation to A-mTBD took place. Additionally, there was no enrichment of the hydrolysis products, H-mTBD-1 (0.001, 0.002, 0.001 kg/h) and H-mTBD-2 (0.000, 0.001, and 0.0004 kg/h) in the residue, and as such, an alternative method needs to be considered for the separation of the hydrolysis products from the IL, as most of it stayed within the distillate. Table 4 shows that >80% of mTBD can be recaptured from the distillate at all the measured flow rates.

**Table 4:** Mass Balance of Individual Components in Experiment 1^a^

component	*ṁ*_feed_ kg/h	*ṁ*_distillate_ kg/h	*ṁ*_residue_ kg/h	*ṁ*_losses_ kg/h
*ṁ* = 0.378 kg/h				
mTBD^+^	0.263	0.220	0.020	0.033
TBD	0.004	0.003	0.000	
H-mTBD-1	0.006	0.005	0.001	
H-mTBD-2	0.003	0.002	0.000	
–OAc	0.103	0.086	0.008	
*ṁ* = 0.766 kg/h				
mTBD^+^	0.533	0.467	0.060	0.0101
TBD	0.007	0.005	0.000	
H-mTBD-1	0.012	0.010	0.002	
H-mTBD-2	0.006	0.005	0.001	
–OAc	0.209	0.183	0.023	
*ṁ* = 0.961 kg/h				
mTBD^+^	0.667	0.566	0.017	0.0232
TBD	0.009	0.006	0.000	
H-mTBD-1	0.015	0.013	0.001	
H-mTBD-2	0.007	0.006	0.000	
–OAc	0.262	0.222	0.005	

a *ṁ*_feed_: mass flow rate of feed, *ṁ*_distillate_: mass flow rate of distillate, *ṁ*_residue_: mass flow rate of residue, *ṁ*_losses_: mass flow rate of losses; *ṁ*_feed_ = *ṁ*_distillate_ + *ṁ*_residue_ + *ṁ*_losses_.

### 3.2 Experiment 2

In experiment 2, four different flow rates (0.378, 0.572, 0.766, and 0.961 kg/h) were investigated at a pressure of 9.2 mbar and at the same temperature settings as experiment 1. The feed components were [mTBDH]­[OAc], H-mTBD-1, H-mTBD-2, NaCl, and KCl. At a lower flow rate of 0.378 kg/h, 97% of the feed is recovered in the distillate. The distillate recovery was higher when the feed flow rate was lower. This conclusion is also supported in Phuong’s work, even though the operating pressure was lower in her work.^16^ It did not affect the overall separation efficiency of the process. After the sample collection, NMR analysis was carried out to determine the distribution of the components in each of the streams. According to the NMR, a portion of the bases stayed unprotonated (0.4 mol %) in the residue in the presence of the salts. A small amount (0.75–0.85%) of A-mTBD was found in the residue for all flow rates within experiment 2 except when the flow rate was 0.572 kg/h, as presented in SI Table S10. AAS analysis was carried out to determine the amount of Na and K in the samples, and the results (SI Table S11) indicated that the enrichment of K and Na takes place in the residue, which meant that these salts can be separated using the SPD unit even though there is slight entrainment into the distillate. Additionally, since the amount of the impurities added was relatively low (0.1% of the feed), chloride was also neglected from the mass balance calculations. From Table S10 of SI, it can be observed that >90% of the expensive base mTBD can be recovered from the distillate.

### 3.3 Experiment 3

Three runs of the experiment were carried out at flow rates of 0.378, 0.766, and 1.155 kg/h, and the recovery of the distillate components was 99.2%, 93.7% and 77.1% by mass, respectively. The NMR analysis of the samples indicated the formation of A-mTBD in the feed sample. The samples of the distillate and residue did not indicate the presence of A-mTBD. Additionally, the samples collected from the traps also did not confirm the presence of A-mTBD. Also, there seemed to be no increase or decrease in the base component of the IL. The results of the ICP analysis provided in SI Table S12 indicate the enrichment of Ca in the residues. At flow rates of 1.155 and 0.766 kg/h, enrichment of Ca takes place in the residue, whereas at a flow rate of 0.378 kg/h, no Ca is detected in the sample. Traces of Ca were also present in the distillate, indicating entrainment to a certain degree in the SPD unit. The enrichment of Ca in the residue is an indication that it can be separated from the IL using the SPD unit to a high degree. Table S13 shows that the recovery of mTBD is >98% when the feed flow rate is 0.378 and 0.766 kg/h, while still having a recovery of >80% when the feed flow rate is 1.155 kg/h.

### 3.4 Experiment 4

Experiment 4 was performed at three different flow rates of 0.378, 0.572, and 0.766 kg/h at an operating pressure of 9.2 mbar as well. The selection of low flow rates of 0.378 and 0.572 kg/h resulted in no accumulation of residue within the two runs. No traces of material were found in the traps as well, even though the feed was not recovered completely in the distillate. This could be associated with an internal holdup within the unit. The NMR spectrum of the feed sample did not confirm the presence of xylan. The analysis of the distillate and residue samples indicated the presence of traces of xylan that were not quantifiable. To further determine the amount of xylan, sulfuric acid hydrolysis of [mTBDH]­[OAc] was carried out following the quantification of the xylose by HPAEC-PAD. At the flow rate of 0.378 kg/h, 99.7% by weight of the feed went to the distillate. Additionally, A-mTBD was detected in the feed at this flow rate. Component mass balance carried out at 0.378 kg/h indicated that no separation of any components occurred from the feed to the distillate. There was a slight decrease in the amount of A-mTBD, from 0.0068 kg/h in the feed to 0.0034 kg/h in the distillate, even though it contained many impurities. Similar results were replicated at a flow rate of 0.572 kg/h, except that A-mTBD is absent from the distillate and undetected in the residue in this case. An observation of note is the absence of H-mTBD-2 in all three runs from the feed. A possible explanation is that xylan is able to draw out water from the decomposition product, H-mTBD-2, resulting in the formation of A-mTBD. An artificial feed mixture was made by using the same experimental composition as the feed to study the presence or absence of xylan in the distillate. The chromatograms obtained from HPAEC-PAD indicated the absence of xylose in the distillates and its presence in the residue. The integration results of the HPAEC-PAD analysis are provided in SI Table S14. From Table S15, at the feed flow rate of 0.378 kg/h, >99% of mTBD can be recovered from the distillate, while at 0.572 and 0.766 kg/h, the recovery is >95%.

### 3.5 Experiment 5

The feed in experiment 5 consisted of [mTBDH]­[OAc], H-mTBD-1, H-mTBD-2, and lactic acid. The operating pressure was 9.14 mbar, and the temperatures remained consistent with the previous runs. Three runs were carried out at flow rates of 0.378, 0.480, and 0.572 kg/h with recovered distillates of 98.1, 95.2, and 98.6 wt %, respectively. The NMR analysis carried out indicated the presence of trace amounts of lactic acid at a flow rate of 0.378 kg/h. Overall, the amount of lactic acid added to the feed (0.1 wt %) is too low to be quantified, even with its presence detected via NMR. Additionally, the NMR peaks of H-mTBD-2 were covered by the peaks of A-mTBD, which made it difficult to quantify them. Also, a slight degradation of the IL took place during this run, as the presence of A-mTBD was observed in the residue at all three runs. Analysis of the samples from the nitrogen trap showed traces of mainly water and mTBD. SI Table S16 shows that >90% of mTBD can be recovered from the distillate at all the measured flow rates. At a feed flow rate of 0.572 kg/h, the mTBD recovery (>98%) is relatively higher than the lower feed flow rate of 0.378 kg/h, where the recovery is >96%.

### 3.6 Experiment 6

In experiment 6, all the impurities (0.1 wt %) from the previous runs were mixed with [mTBDH]­[OAc] and H-mTBD-1, and H-mTBD-2. The experimental runs were operated at 9.14 mbar at three different flow rates of 0.572, 0.480, and 0.378 kg/h. No residue was obtained at the flow rate of 0.378 kg/h. The analysis of the samples was carried out using NMR, AAS, and ICP. Xylan and lactic acid could be observed in the NMR spectrum and quantified in the residue samples, but it was difficult to quantify them in the feed samples. The AAS and ICP analyses of the salts indicated that the enrichment of K, Na, and Ca took place in the residue. The analysis also indicates that traces of Na and Ca were detected in the distillate, while K was below the limit of quantification. Even though the amount of salt added to the feed was low (0.1 wt %), the presence of the salts in the distillate is enough to confirm entrainment. The decomposition product, H-mTBD-2, seems to be consumed in the feed by interacting with one of the impurities (lactic acid or xylan) in the mixture. This is supported by experiments 4 and 5, wherein H-mTBD-2 was consumed in the feed, even though 2% by mass of the hydrolysis products formed part of the overall feed. Table S17 shows that the recovery of mTBD is >90% in the distillate at the measured flow rates.

### 3.7 Experiment 7

Experiment 7 is carried out under the same conditions as experiment 6. The only difference is the increase of impurities (0.3 wt %) added to the feed. The flow rates were set at 0.572, 0.868, and 1.063 kg/h to be able to detect which components of the feed would be obtained in the residue. Low flow rates, while providing recovery of most of the components in the distillate, did not provide tangible results with respect to the components obtained in the residue. AAS and ICP analyses carried out on the samples indicated the enrichment of the ions K, Na, and Ca in the residue. No A-mTBD formation was detected in the feed, distillate, or residue. The decomposition product H-mTBD-2 is absent/undetected in the feed. The NMR analysis carried out on the hydrolysis product sample indicated the presence of H-mTBD-2 prior to its addition to the overall feed. This further supports the interaction and consumption of H-mTBD-2 with one of the added impurities within the feed. At a flow rate of 0.572 kg/h, all impurities can be seen, but lactic acid and xylan could not be quantified via NMR. Additionally, from this experimental set, it is observed that 0.1% of the feed seemed like a low amount of impurities to be able to obtain quantifiable results for xylan and lactic acid. The heavier impurities, such as xylan and lactic acid, undergo separation, as they are enriched in the residue. At a flow rate of 1.06 kg/h, xylan is detected in the residue but cannot be quantified. NMR analysis also indicated that xylan is missing in the feed. A possible reason for this is that the mixture is not homogeneous. In all cases, the chloride is assumed to be bound to the salts, as they have not been analyzed. K was below detection limits in the distillate at flow rates of 0.86 and 1.06 kg/h. It can be observed at a flow rate of 1.062 kg/h; the distillate has only mTBD, TBD, and acetic acid (HOAc) components. Apart from the inorganic salt content, there is no presence of any decomposition products. This NMR analysis closely matches the pure IL analytics, except that the salts are present. In the residue, enrichment of K, Na, and Cl occurs, indicating that the salts can be separated from the feed. Table 5 shows that at 0.572 kg/h, >90% of the mTBD can be recovered, while at flow rates of 0.868 and 1.063 kg/h, >75% of the mTBD can be obtained in the distillate.

**Table 5:** Mass Balance of Individual Components in Experiment 7^a^

component	*ṁ*_feed_ kg/h	*ṁ*_distillate_ kg/h	*ṁ*_residue_ kg/h	*ṁ*_losses_ kg/h
*ṁ* = 0.572 kg/h				
mTBD^+^	0.397	0.368	0.002	0.002
TBD	0.005	0.004	2.86 × 10^–5^	
H-mTBD-1	0.009	0.008	0.000	
H-mTBD-2	0.000	0.037	7.04 × 10^–5^	
–OAc	0.156	0.144	0.001	
K	0.000	1.16 × 10^–6^	6.96 × 10^–6^	
Na	0.000	3.68 × 10^–6^	1.63 × 10^–5^	
Ca	4.62 × 10^–5^	3.79 × 10^–6^	2.08 × 10^–5^	
xylan			0.002	
lactic acid			9.88 × 10^–5^	
*ṁ* = 0.868 kg/h				
mTBD^+^	0.603	0.490	0.058	0.009
TBD	0.008	0.007	0.002	
H-mTBD-1	0.013	0.011	0.004	
H-mTBD-2	0.000	0.049	0.004	
–OAc	0.236	0.192	0.023	
K	2.00 × 10^–4^	0.000	3.94 × 10^–4^	
Na	3.68 × 10^–4^	7.44 × 10^–6^	3.73 × 10^–4^	
Ca	7.02 × 10^–5^	3.46 × 10^–6^	3.63 × 10^–4^	
xylan			0.009	
lactic acid	0.003		0.008	
*ṁ* = 1.063 kg/h				
mTBD^+^	0.738	0.577	0.109	0.078
TBD	0.010	0.008	0.002	
H-mTBD-1	0.016	0.000	0.005	
H-mTBD-2	0.000	0.000	0.002	
–OAc	0.289	0.226	0.043	
K	2.45 × 10^–4^		3.30 × 10^–4^	
Na	4.51 × 10^–4^	2.98 × 10^–5^	3.09 × 10^–4^	
Ca	8.59 × 10^–5^	1.32 × 10^–5^	3.28 × 10^–4^	
xylan				
lactic acid	0.00434		0.0076	

a *ṁ*_feed_: mass flow rate of feed, *ṁ*_distillate_: mass flow rate of distillate, *ṁ*_residue_: mass flow rate of residue, *ṁ*_losses_: mass flow rate of losses; *ṁ*_feed_ = *ṁ*_distillate_ + *ṁ*_residue_ + *ṁ*_losses_.

### 3.8 Experiment 8

The contents of the feed in this experiment were adjusted based on the results of the previous experimental runs. Three different flow rates of the run are carried out at 1.155, 1.063, and 0.961 kg/h at 9 mbar operating pressure. One of the goals of this work was to find the conditions under which A-mTBD formation is initiated; hence, the evaporator temperature was raised to 220 °C. Also, the adjustments were carried out to adapt to the conditions like those of the recovery step of the solvent in the Ioncell process. Additionally, the salts NaCl and KCl were excluded from this experimental run as the study of the behavior of bivalent cations such as CaCl_2_ seemed more interesting due to their interaction with the base mTBD. Ions having high charge density such as calcium could trigger ion–exchange reactions by overcoming the polarity of the IL, which in turn would modify its behavior.^21^ In the presence of water, inorganics could interact with water, resulting in a salting-in/-out phenomenon for the IL.^21^ Additionally, apart from the hydrolysis product, water was added to the feed, as this would give the composition of the ice trap contents. The ice trap contents could not be quantified in previous run conditions due to the lack of water content in the feed. Xylan is not included in this run, as the expected behavior is that it precipitates during spinning in the coagulation bath, even though it dissolves at a moderate concentration in the IL.^18^ The presence of high contents of TBD in mTBD did not negatively affect the cellulose dissolution process.^41^ Therefore, it is not addressed closely in this work, apart from the feed containing a relatively low amount of TBD with respect to mTBD. The NMR results indicated that A-mTBD forms when the feed is mixed, but no formation of A-mTBD took place in the SPD, even in the presence of CaCl_2_ and lactic acid. Consequently, the experiment needs to be carried out at an evaporator temperature higher than 220 °C. Based on the vapor pressure correlation in Figure 2, it is possible to reach even higher pressures using the current setup. From Table 6, it can be observed that more than 75% of mTBD is recovered in the distillate at all the measured flow rates.

**Table 6:** Mass Balance of Individual Components in Experiment 8

component	^a^ *ṁ*_feed_ kg/h	^a^ *ṁ*_distillate_ kg/h	^a^ *ṁ*_residue_ kg/h	^a^ *ṁ*_losses_ kg/h
*ṁ* = 1.063 kg/h				
mTBD^+^	0.739	0.567	0.094	0.078
TBD	0.000	0.006	0.002	
H-mTBD-1	0.012	0.020	0.004	
H-mTBD-2	0.001	0.001	0.001	
–OAc	0.307	0.240	0.043	
Ca	2.57 × 10^–4^	3.73 × 10^–5^	2.82 × 10^–4^	
lactic acid	0.002	0.001	0.004	
*ṁ* = 0.961 kg/h				
mTBD^+^	0.668	0.504	0.062	0.113
TBD	0.000	0.007	0.001	
H-mTBD-1	0.011	0.019	0.003	
H-mTBD-2	0.001	0.001	0.001	
–OAc	0.278	0.218	0.027	
Ca	2.32 × 10^–4^	2.74 × 10^–5^	2.50 × 10^–4^	
lactic acid	0.002	0.001	0.003	
*ṁ* = 1.155 kg/h				
mTBD^+^	0.803	0.603	0.108	0.0915
TBD	0.000	0.009	0.002	
H-mTBD-1	0.013	0.023	0.005	
H-mTBD-2	0.009	0.002	0.001	
–OAc	0.334	0.252	0.052	
Ca	2.79 × 10^–4^	1.07 × 10^–4^	2.55 × 10^–4^	
lactic acid	0.002	0.001	0.003	

a *ṁ*_feed_: mass flow rate of feed, *ṁ*_distillate_: mass flow rate of distillate, *ṁ*_residue_: mass flow rate of residue, *ṁ*_losses_: mass flow rate of losses; *ṁ*_feed_ = *ṁ*_distillate_ + *ṁ*_residue_ + *ṁ*_losses_.

Overall, there seemed to be less interference and synergistic effects on comparing experiments with single impurities to those with multiple contaminants. The impurities remained largely consistent for the most part, and no significant deviations beyond experimental variability were detected. Addition of Ca (experiment 3) and xylan (experiment 4) impurities to the IL did result in the degradation of the hydrolysis products further to A-mTBD. In the multiple contaminant experiments, this degradation has not been observed. An observation in experiments 4 to 8 indicated that the presence of xylan and lactic acid prevents the degradation of the IL to H-mTBD-2. This suggests that the SPD process is relatively resilient to the presence of multiple impurities, but at the same time, more detailed, targeted studies are needed to definitively assess potential interactions, particularly at higher impurity concentrations. This highlights a valuable direction for future work to further investigate potential compound-specific or concentration-dependent effects that might emerge under varied processing conditions.

For further studies, it is recommended to consider textile dyes,^25^ residual pulping chemicals,^26^ and finishing agents, in the analysis of the recycled feed, as it would develop the separation process and ensure higher purity of the IL while providing improved quality in the final product.

## 4 Conclusions

Within this study, the conditions needed to purify [mTBDH]­[OAc] in the presence of different impurities via SPD are illustrated. The setup for carrying out the SPD experiments was optimized, and the temperature at which A-mTBD formation takes place in the presence of certain impurities was determined. The overall results of the experiments confirmed that the feed containing the impurities K, Na, Ca, xylan, and lactic acid can be purified into a clean distillate using an SPD unit. In short, the results of the experiments confirmed that the base mTBD can be recovered in almost all cases. High recovery fractions are required for possible future industrial applications, which are realized by reduced flow rates. This work also provides the pressure ranges to carry out the purification to achieve maximum recovery of the base mTBD. Additionally, the formation of A-mTBD was inhibited by keeping the residence time at high temperature short. Experiments 1–5 concluded that the purification of IL from the individual impurities is possible. In experiments 6–8, the mixture of impurities coming into the IL during the pulping stage of the ioncell process was separated. In summary, the presence of multiple contaminants showed less interference compared with single impurities, with no significant deviations beyond experimental variability. Crucially, xylan and lactic acid appeared to inhibit degradation of the IL to H-mTBD-2, indicating possible stabilizing effects in both single and multiple contaminant systems. Overall, the experimental work and analytical results give an understanding of what happens to [mTBDH]­[OAc] in the presence of the studied impurities in an SPD unit. These results can be used for the design of a purification process of high quantities of [mTBDH]­[OAc].

## Supplementary Material



## Data Availability

The data that support the findings of this study are contained within the manuscript.

## Notes

Mass balance of SPD from all experiments, analytics of the samples using NMR, AAS, ICP, and HPAEC-PAD, and compositional mass balance of experiments 2–6 (PDF)
