# Supplementary material for: Purification of Ionic Liquid [mTBDH][OAc] Utilizing the Short-Path Distillation Technique
Source: ACS Omega. 2025 Sep 9;10(37):42632–43. doi: 10.1021/acsomega.5c04442 (PMC12461294; doi:10.1021/acsomega.5c04442)
Supplement: Supplementary file 1 [file ao5c04442_si_001.pdf]

*Supporting Information*

# Purification of ionic liquid [mTBDH][OAc] utilizing short-path distillation technique

Mohammed Saad[a,*], Inge Schlapp-Hackl[b], Petri Uusi-Kyyny[a], Ville Alopaeus[a]

[a]Aalto University, School of Chemical Engineering, Department of Chemical and Metallurgical Engineering, P.O. Box 11000, FI-00076 Aalto, Finland

[b]Aalto University, School of Chemical Engineering, Department of Bioproducts and Biosystems, P.O. Box 11000, FI-00076 Aalto, Finland

[*]Corresponding author; Email: mohammed.saad@aalto.fi

Table S1.NMR analysis of samples from experiment 1.

| Type | $mTBD^+$ [mole%] | H-mTBD-2 [mole%] | TBD [mole%] | H-mTBD-1 [mole%] | $^-OAc$ [mole%] |
|---|---|---|---|---|---|
| Feed | 48.9 | 0.5 | 0.7 | 1.0 | 48.9 |
| Distillate 1 | 48.9 | 0.5 | 0.7 | 1.0 | 48.9 |
| Residue 1 | 48.5 | 1.0 | 0.5 | 1.5 | 48.5 |
| Distillate 2 | 49.0 | 0.5 | 0.6 | 1.0 | 49.0 |
| Residue 2 | 48.8 | 0.5 | 0.4 | 1.5 | 48.8 |
| Distillate 3 | 49.0 | 0.5 | 0.6 | 1.0 | 49.0 |
| Residue 3 | 42.5 | 0.4 | 0.6 | 1.7 | 54.8 |
| $N_2$ trap | 49.7 | - | - | - | 50.3 |
| Ice trap | 55.8 | 1.1 | 0.6 | 2.2 | 40.2 |

Table S2.NMR analysis of samples from experiment 2.

| Sample | $mTBD^+$ [mole%] | A-mTBD [mole%] | H-mTBD-2 [mole%] | $mTBD^-$ [mole%] | TBD [mole%] | H-mTBD-1 [mole%] | $^-OAc$ [mole%] |
|---|---|---|---|---|---|---|---|
| Feed | 48.3 | - | 0.5 | - | 0.9 | 1.9 | 48.3 |
| Distillate 1 | 48.0 | - | 0.5 | - | 0.7 | 2.9 | 48.0 |
| Distillate 2 | 48.0 | - | 0.5 | - | 0.5 | 2.9 | 48.0 |
| Distillate 3 | 47.8 | - | 1.0 | - | 0.5 | 2.9 | 47.8 |
| Distillate 4 | 48.0 | - | 0.5 | - | 0.7 | 2.9 | 48.0 |
| Residue 1 | 39.5 | 0.4 | 0.8 | - | 0.4 | 3.6 | 55.3 |
| Residue 2 | 43.8 | 0.4 | 1.3 | 0.4 | 0.5 | 4.4 | 49.1 |
| Residue 3 | 41.1 | 0.8 | 1.2 | 0.4 | 0.5 | 4.9 | 51.0 |
| Residue 4 | 46.9 | - | 1.4 | - | 0.5 | 4.2 | 46.9 |

Table S3. NMR analysis of samples from experiment 3.

| Sample | $mTBD^+$ [mole%] | A-mTBD [mole%] | H-mTBD-2 [mole%] | TBD [mole%] | H-mTBD-1 [mole%] | $^-OAc$ [mole%] |
|---|---|---|---|---|---|---|
| Distillate 1 | 49.0 | - | 0.5 | 0.6 | 1.0 | 49.0 |

| | | | | | | |
|---|---|---|---|---|---|---|
| Distillate 2 | 49.0 | - | 0.5 | 0.6 | 1.0 | 49.0 |
| Distillate 3 | 49.0 | - | 0.5 | 0.6 | 1.0 | 49.0 |
| Residue 1 | 43.6 | - | 0.4 | 0.5 | 1.7 | 53.7 |
| Residue 2 | 46.5 | - | 0.5 | 0.5 | 1.4 | 51.1 |
| Residue 3 | 44.0 | - | 0.4 | 0.5 | 1.3 | 53.7 |
| Feed | 46.7 | 0.9 | 0.9 | 0.5 | 1.4 | 49.5 |
| Pure IL | 49.7 | - | - | 0.6 | - | 49.7 |

Table S4. NMR analysis of samples from experiment 4.

| Sample type | $mTBD^+$ [mole%] | A-mTBD [mole%] | H-mTBD-2 [mole%] | TBD [mole%] | H-mTBD-1 [mole%] | $^-$OAc [mole%] |
|---|---|---|---|---|---|---|
| Distillate 1 | 45.43 | 0.45 | - | 0.51 | 1.82 | 51.79 |
| Distillate 2 | 46.06 | - | - | 0.52 | 1.84 | 51.58 |
| Distillate 3 | 45.43 | - | - | 0.51 | 1.82 | 52.24 |
| Residue 2 | 34.59 | 0.69 | 0.69 | 0.39 | 2.08 | 61.57 |
| Residue 3 | 44.30 | 0.89 | 1.33 | 0.33 | 2.66 | 50.50 |
| Feed | 45.02 | 0.90 | - | 0.51 | 1.80 | 51.77 |
| Pure IL | 49.63 | - | - | 0.74 | - | 49.63 |

Table S5. NMR analysis of samples from experiment 5.

| Sample type | $mTBD^+$ [mole%] | A-mTBD [mole%] | H-mTBD-2 [mole%] | TBD [mole%] | H-mTBD-1 [mole%] | $^-$OAc [mole%] | Lactic acid |
|---|---|---|---|---|---|---|---|
| Pure IL | 49.63 | - | - | 0.74 | - | 49.63 | - |
| Feed | 49.14 | - | - | 0.74 | 0.98 | 49.14 | - |
| Distillate 1 | 49.23 | - | - | 0.55 | 0.98 | 49.23 | - |
| Distillate 2 | 49.23 | - | - | 0.55 | 0.98 | 49.23 | - |
| Distillate 3 | 49.23 | - | - | 0.55 | 0.98 | 49.23 | - |
| Residue 1 | 41.54 | 1.66 | 2.08 | 0.31 | 3.32 | 51.09 | - |
| Residue 2 | 46.48 | 0.02 | 2.32 | 0.52 | 3.25 | 47.41 | - |
| Residue 3 | 46.92 | 0.46 | 2.35 | 0.53 | 2.82 | 46.92 | 0.47 |

Table S6. NMR analysis of samples from experiment 6.

| Sample type | $mTBD^+$ [mole%] | H-mTBD-2 [mole%] | TBD [mole%] | H-mTBD-1 [mole%] | $^-OAc$ [mole%] | Lactic acid | Xylan |
|---|---|---|---|---|---|---|---|
| Pure IL | 49.72 | - | 0.56 | - | 49.72 | - | - |
| Feed | 49.23 | - | 0.55 | 0.98 | 49.23 | - | - |
| Distillate 1 | 49.23 | - | 0.55 | 0.98 | 49.23 | - | - |
| Distillate 2 | 49.23 | - | 0.55 | 0.98 | 49.23 | - | - |
| Distillate 3 | 48.99 | - | 0.55 | 1.47 | 48.99 | - | - |
| Residue 1 | 46.97 | 1.41 | 0.70 | 1.88 | 46.97 | 0.94 | 1.13 |
| Residue 2 | 46.56 | 1.40 | 0.87 | 1.86 | 46.56 | 1.40 | 1.35 |
| Residue 3 | 46.80 | 0.94 | 0.88 | 1.87 | 46.80 | 1.40 | 1.31 |

Table S7. NMR analysis of samples from experiment #7.

| Sample type | $mTBD^+$ [mole%] | H-mTBD-2 [mole%] | TBD [mole%] | H-mTBD-1 [mole%] | $^-OAc$ [mole%] | Lactic acid | Xylan |
|---|---|---|---|---|---|---|---|
| Pure IL | 49.72 | - | 0.56 | - | 49.72 | - | - |
| Hydrolysis product | 0.00 | 8.26 | - | 91.74 | - | - | - |
| Feed | 48.90 | - | 0.73 | 0.98 | 48.90 | 0.49 | - |
| Distillate 1 | 47.14 | 4.24 | 0.53 | 0.94 | 47.14 | - | - |
| Distillate 2 | 47.06 | 4.24 | 0.71 | 0.94 | 47.06 | - | - |
| Distillate 3 | 49.63 | - | 0.74 | - | 49.63 | - | - |
| Residue 1 | 29.46 | 0.88 | 0.44 | 3.53 | 29.46 | 2.36 | 33.87 |
| Residue 2 | 39.17 | 2.74 | 1.18 | 2.35 | 39.17 | 9.01 | 6.38 |
| Residue 3 | 45.40 | 0.91 | 1.02 | 1.82 | 45.40 | 5.45 | - |

Table S8. NMR analysis of samples from experiment 8.

| Sample type | $mTBD^+$ [mole%] | H-mTBD-2 [mole%] | $mTBD^-$ [mole%] | TBD [mole%] | H-mTBD-1 [mole%] | $^-OAc$ [mole%] | lactic acid [mole%] |
|---|---|---|---|---|---|---|---|
| Distillate 1 dark | 46.1 | 0.05 | - | 0.83 | 1.43 | 51.54 | 0.05 |

| | | | | | | | |
|---|---|---|---|---|---|---|---|
| Distillate 1 light | 47.14 | 0.15 | - | 0.82 | 1.6 | 50.15 | 0.15 |
| Residue 1 | 42.73 | 0.3 | - | 0.74 | 1.92 | 52 | 2.31 |
| Distillate 2 | 46.93 | 0.05 | - | 0.56 | 1.5 | 50.82 | 0.14 |
| Residue 2 | 43.43 | 0.43 | - | 0.98 | 1.74 | 50.38 | 3.04 |
| Distillate 3 | 46.34 | 0.09 | - | 0.73 | 1.58 | 51.03 | 0.23 |
| Residue 3 | 44.54 | 0.45 | - | 0.67 | 1.78 | 49.44 | 3.12 |
| Ice trap | - | 11.83 | 34.41 | - | 53.76 | - | - |
| Feed | 48.05 | 0.05 | - | - | 0.72 | 50.99 | 0.19 |

Table S9. SPD results of all experiments at $p$ = 9.14 mbar.

| $\dot{m}^a$ (kg/h) | $m_{distillate}$[a] (wt%) | $m_{residue}$[a] (wt%) | $m_{losses}$[a] (wt%) |
|---|---|---|---|
| Experiment 1: IL + Hydrolysis product | | | |
| 0.766 | 83.5 | 7.8 | 8.7 |
| 0.961 | 87.4 | 11.2 | 1.3 |
| 1.100 | 85 | 13 | 2 |
| Experiment 2: IL + Hydrolysis product + NaCl +KCl | | | |
| 0.378 | 97 | 1 | 2 |
| 0.572 | 92 | 3 | 5 |
| 0.766 | 92 | 5 | 3 |
| 0.961 | 83 | 13 | 4 |
| Experiment 3: IL + Hydrolysis product + $CaCl_2$ | | | |
| 0.378 | 99.2 | 0.5 | 0.3 |
| 0.766 | 93.7 | 5.1 | 1.3 |
| 1.155 | 77.1 | 15.2 | 0.76 |
| Experiment 4: IL + Hydrolysis product + xylan | | | |
| 0.378 | 99.7 | 0 | 0.3 |
| 0.572 | 95.5 | 0 | 4.4 |
| 0.766 | 93.7 | 1.4 | 4.8 |
| Experiment 5: IL + Hydrolysis product + lactic acid | | | |
| 0.378 | 98.1 | 1.1 | 0.8 |

| | | | |
|---|---|---|---|
| 0.480 | 95.2 | 1.5 | 3.3 |
| 0.572 | 98.6 | 1.2 | 0.2 |
| Experiment 6: IL + Hydrolysis product + NaCl +KCl + $CaCl_2$ + xylan + lactic acid | | | |
| 0.378 | 93.9 | 0 | 6.1 |
| 0.480 | 97.5 | 2.1 | 0.4 |
| 0.572 | 96 | 1.6 | 2.6 |
| Experiment 7: IL + Hydrolysis product + NaCl +KCl + $CaCl_2$ + xylan + lactic acid | | | |
| 0.572 | 98.6 | 1 | 0.4 |
| 0.868 | 86.6 | 12.4 | 1.0 |
| 1.063 | 76.7 | 15.9 | 7.4 |
| Experiment 8*: IL + Hydrolysis product + $CaCl_2$ + $H_2O$ + lactic acid | | | |
| 0.961 | 78 | 10 | 12 |
| 1.063 | 79 | 14 | 7 |
| 1.155 | 77 | 15 | 8 |

$T_{feed}$ = 95°C; $T_{evaporator}$ = 200°C; $T_{condenser}$ =100°C; $T_{distillate}$=100°C; *$T_{evaporator}$ = 220°C.

[a] $\dot{m}$: mass flowrate, $m_{distillate}$: mass of distillate, $m_{residue}$: mass of residue, $m_{loss}$: mass of losses

Table S10. Mass balance of individual components in experiment 2.

| Component | $\dot{m}_{feed}$ kg/h | $\dot{m}_{distillate}$ kg/h | $\dot{m}_{residue}$ kg/h | $\dot{m}_{losses}$ kg/h |
|---|---|---|---|---|
| $\dot{m}$ = 0.378 kg/h | | | | |
| $mTBD^+$ | 0.258 | 0.246 | 0.002 | |
| TBD | 0.004 | 0.003 | - | |
| H-mTBD-1 | 0.011 | 0.016 | - | |
| H-mTBD-2 | 0.003 | 0.003 | - | 0.009 |
| $^-OAc$ | 0.101 | 0.096 | 0.001 | |
| A-mTBD | - | - | 2.3E-06 | |
| K | 2.00E-04 | 1.35E-06 | 6.25E-07 | |
| Na | 1.50E-04 | 1.51E-05 | 2.42E-06 | |
| $\dot{m}$ = 0.572 kg/h | | | | |
| $mTBD^+$ | 0.390 | 0.354 | 0.013 | 0.026 |
| TBD | 0.007 | 0.005 | - | |

| | | | | |
|---|---|---|---|---|
| H-mTBD-1 | 0.017 | 0.024 | 0.001 | |
| H-mTBD-2 | 0.004 | 0.004 | - | |
| $^{-}$OAc | 0.153 | 0.139 | 0.005 | |
| A-mTBD | - | - | - | |
| K | 3.02E-04 | 2.23E-06 | 2.20E-06 | |
| Na | 2.27E-04 | 2.52E-05 | 8.26E-06 | |
| $\dot{m}$ = 0.766 kg/h | | | | |
| $mTBD^{+}$ | 0.523 | 0.473 | 0.026 | |
| TBD | 0.009 | 0.005 | - | |
| H-mTBD-1 | 0.023 | 0.032 | 0.003 | |
| H-mTBD-2 | 0.006 | 0.005 | 0.001 | 0.023 |
| $^{-}$OAc | 0.205 | 0.185 | 0.011 | |
| A-mTBD | - | - | - | |
| K | 4.05E-04 | 3.65E-06 | 2.58E-06 | |
| Na | 3.04E-04 | 6.07E-05 | 1.26E-05 | |
| $\dot{m}$ = 0.961 kg/h | | | | |
| $mTBD^{+}$ | 0.655 | 0.535 | 0.072 | |
| TBD | 0.011 | 0.005 | 0.001 | |
| H-mTBD-1 | 0.029 | 0.036 | 0.001 | |
| H-mTBD-2 | 0.007 | 0.012 | 0.002 | 0.04 |
| $^{-}$OAc | 0.257 | 0.201 | 0.035 | |
| A-mTBD | - | - | 0.002 | |
| K | 5.08E-04 | 3.57E-06 | 2.26E-05 | |
| Na | 3.81E-04 | 6.03E-05 | 6.63E-05 | |

$\dot{m}_{feed}$: mass flowrate of feed, $\dot{m}_{distillate}$: mass flowrate of distillate, $\dot{m}_{residue}$: mass flowrate of residue, $\dot{m}_{losses}$: mass flowrate of losses; $\dot{m}_{feed} = \dot{m}_{distillate} + \dot{m}_{residue} + \dot{m}_{losses}$.

Table S11. AAS analysis of K and Na in different experiments.

| Sample | Distillate (ppm) | | Residue (ppm) | | Split fraction | |
|---|---|---|---|---|---|---|
| | K | Na | K | Na | K | Na |
| Experiment 2: IL + Hydrolysis product + NaCl +KCl | | | | | | |
| 1 | 3.69 | 41.2 | 226 | 874 | 0.016 | 0.047 |
| 2 | 5.20 | 86.5 | 61 | 298 | 0.085 | 0.291 |
| 3 | 4.47 | 75.5 | 186 | 544 | 0.024 | 0.139 |
| 4 | 4.23 | 47.9 | 110 | 411 | 0.039 | 0.117 |
| Experiment 6: IL + Hydrolysis product + NaCl +KCl + $CaCl_2$ + xylan + lactic acid | | | | | | |
| 1 | <LOQ | 2.82 | 170.60 | 683.31 | 0.00 | 0.0041 |
| 2 | <LOQ | 1.36 | 742.81 | 1330.82 | 0.00 | 0.0010 |
| 3 | <LOQ | 1.29 | 1198.10 | 2403.61 | 0.00 | 0.0005 |
| Experiment 7: IL + Hydrolysis product + NaCl +KCl + $CaCl_2$ + xylan + lactic acid | | | | | | |
| 1 | 2.06 | 6.52 | 1201 | 2817 | 0.002 | 0.0023 |
| 2 | <LOQ | 9.89 | 3667 | 3470 | 0.000 | 0.0028 |
| 3 | <LOQ | 36.54 | 1950 | 1826 | 0.000 | 0.0200 |

Table S12. ICP analysis of Ca in different experiments.

| Sample | Distillate (ppm) | Residue (ppm) | Split fraction |
|---|---|---|---|
| Experiment 3: IL + Hydrolysis product + $CaCl_2$ | | | |
| 1 | 95.95 | 695.05 | 0.138 |
| 2 | 21.26 | 2580.40 | 0.008 |
| 3 | 29.93 | N/A | No residue |
| Experiment 7: IL + Hydrolysis product + NaCl +KCl + $CaCl_2$ + xylan + lactic acid | | | |
| 1 | 6.72 | 3599 | 0.0018 |
| 2 | 4.60 | 3379 | 0.0013 |
| 3 | 16.14 | 1936 | 0.0083 |
| Experiment 8: IL + Hydrolysis product + $CaCl_2$ + $H_2O$ + lactic acid | | | |
| 1 | 52.15 | 1485 | 0.035 |
| 2 | 44.64 | 1911 | 0.023 |
| 3 | 36.41 | 2602 | 0.014 |

Table S13. Mass balance of individual components in experiment 3.

| Component | $\dot{m}_{feed}$ kg/h | $\dot{m}_{distillate}$ kg/h | $\dot{m}_{residue}$ kg/h | $\dot{m}_{losses}$ kg/h |
|---|---|---|---|---|
| $\dot{m}$ = 0.378 kg/h | | | | |
| $mTBD^+$ | 0.250 | 0.261 | 0.001 | 0.001 |
| TBD | 0.002 | 0.003 | 1.09E-05 | |
| H-mTBD-1 | 0.008 | 0.006 | 3.58E-05 | |

| | | | | |
|---|---|---|---|---|
| H-mTBD-2 | 0.005 | 0.003 | 1.19E-05 | |
| $^{-}$OAc | 0.104 | 0.102 | 0.001 | |
| A-mTBD | 0.007 | - | - | |
| Ca | 1.35E-04 | 1.12E-05 | - | |
| $\dot{m}$ = 0.766 kg/h | | | | |
| mTBD$^{+}$ | 0.507 | 0.499 | 0.026 | |
| TBD | 0.005 | 0.005 | - | |
| H-mTBD-1 | 0.017 | 0.011 | 0.001 | 0.01 |
| H-mTBD-2 | 0.011 | 0.005 | - | |
| $^{-}$OAc | 0.210 | 0.196 | 0.011 | |
| A-mTBD | 0.014 | - | - | |
| Ca | 2.74E-04 | 1.53E-05 | 1.01E-04 | |
| $\dot{m}$ = 1.155 kg/h | | | | |
| mTBD$^{+}$ | 0.764 | 0.620 | 0.113 | |
| TBD | 0.008 | 0.006 | 0.001 | |
| H-mTBD-1 | 0.026 | 0.014 | 0.005 | 0.088 |
| H-mTBD-2 | 0.017 | 0.007 | 0.001 | |
| $^{-}$OAc | 0.318 | 0.243 | 0.055 | |
| A-mTBD | 0.021 | - | - | |
| Ca | 4.12E-04 | 8.55E-05 | 1.22E-04 | |

$\dot{m}_{feed}$: mass flowrate of feed, $\dot{m}_{distillate}$: mass flowrate of distillate, $\dot{m}_{residue}$: mass flowrate of residue, $\dot{m}_{losses}$: mass flowrate of losses;
$\dot{m}_{feed} = \dot{m}_{distillate} + \dot{m}_{residue} + \dot{m}_{losses}$.

Table S14. HPAEC-PAD results of residue from experiment 4.

| No. | Peak Name | Retention Time (min) | Area nC*min | Height nC | Relative Area % | Relative Height % | Amount mg/l |
|---|---|---|---|---|---|---|---|
| 1 | Glucose | 15.509 | 0.656 | 0.998 | 3.12 | 3.42 | 26.071 |
| 2 | Xylose | 18.684 | 20.397 | 28.207 | 96.88 | 96.58 | 793.566 |
| Total: | | | 21.054 | 29.205 | 100.00 | 100.00 | |

Table S15. Mass balance of individual components in experiment 4.

| Component | $\dot{m}_{feed}$ kg/h | $\dot{m}_{distillate}$ kg/h | $\dot{m}_{residue}$ kg/h | $\dot{m}_{losses}$ kg/h |
|---|---|---|---|---|
| | | $\dot{m}$ = 0.378 kg/h | | |
| $mTBD^+$ | 0.246 | 0.248 | - | |
| TBD | 0.002 | 0.002 | - | |
| H-mTBD-1 | 0.011 | 0.011 | - | 0.001 |
| H-mTBD-2 | - | - | - | |
| $^-OAc$ | 0.111 | 0.111 | - | |
| A-mTBD | 0.007 | 0.003 | - | |
| | | $\dot{m}$ = 0.572 kg/h | | |
| $mTBD^+$ | 0.373 | 0.366 | - | |
| TBD | 0.004 | 0.004 | - | |
| H-mTBD-1 | 0.017 | 0.016 | - | 0.025 |
| H-mTBD-2 | - | - | - | |
| $^-OAc$ | 0.168 | 0.161 | - | |
| A-mTBD | 0.010 | - | - | |
| | | $\dot{m}$ = 0.766 kg/h | | |
| $mTBD^+$ | 0.500 | 0.477 | 0.007 | |
| TBD | 0.005 | 0.005 | - | |
| H-mTBD-1 | 0.022 | 0.021 | - | 0.037 |
| H-mTBD-2 | - | - | - | |
| $^-OAc$ | 0.225 | 0.215 | 0.003 | |
| A-mTBD | 0.014 | - | 0.014 | |

$\dot{m}_{feed}$: mass flowrate of feed, $\dot{m}_{distillate}$: mass flowrate of distillate, $\dot{m}_{residue}$: mass flowrate of residue, $\dot{m}_{losses}$: mass flowrate of losses; $\dot{m}_{feed} = \dot{m}_{distillate} + \dot{m}_{residue} + \dot{m}_{losses}$.

Table S16. Mass balance of individual components in experiment 5.

| Component | $\dot{m}_{feed}$ kg/h | $\dot{m}_{distillate}$ kg/h | $\dot{m}_{residue}$ kg/h | $\dot{m}_{losses}$ kg/h |
|---|---|---|---|---|
| | | $\dot{m}$ = 0.378 kg/h | | |

| | | | | |
|---|---|---|---|---|
| $mTBD^+$ | 0.269 | 0.260 | 0.002 | 0.003 |
| TBD | 0.004 | 0.003 | - | |
| H-mTBD-1 | - | 0.006 | - | |
| H-mTBD-2 | - | - | - | |
| $^-OAc$ | 0.105 | 0.102 | - | |
| A-mTBD | - | - | - | |
| Lactic acid | - | - | - | |
| $\dot{m}$ = 0.480 kg/h | | | | |
| $mTBD^+$ | 0.341 | 0.320 | 0.005 | 0.016 |
| TBD | 0.005 | 0.003 | - | |
| H-mTBD-1 | - | 0.007 | - | |
| H-mTBD-2 | - | - | - | |
| $^-OAc$ | 0.134 | 0.126 | 0.002 | |
| A-mTBD | - | - | - | |
| $\dot{m}$ = 0.572 kg/h | | | | |
| $mTBD^+$ | 0.400 | 0.395 | 0.004 | 0.001 |
| TBD | 0.005 | 0.004 | - | |
| H-mTBD-1 | 0.009 | 0.009 | - | |
| H-mTBD-2 | - | - | - | |
| $^-OAc$ | 0.157 | 0.155 | 0.002 | |
| A-mTBD | - | - | - | |

$\dot{m}_{feed}$: mass flowrate of feed, $\dot{m}_{distillate}$: mass flowrate of distillate, $\dot{m}_{residue}$: mass flowrate of residue, $\dot{m}_{losses}$: mass flowrate of losses; $\dot{m}_{feed} = \dot{m}_{distillate} + \dot{m}_{residue} + \dot{m}_{losses}$.

Table S17. Mass balance of individual components in experiment 6.

| Component | $\dot{m}_{feed}$ kg/h | $\dot{m}_{distillate}$ kg/h | $\dot{m}_{residue}$ kg/h | $\dot{m}_{losses}$ kg/h |
|---|---|---|---|---|
| $\dot{m}$ = 0.572 kg/h | | | | |
| $mTBD^+$ | 0.401 | 0.384 | 0.006 | 0.015 |
| TBD | 0.004 | 0.004 | 7.76E-05 | |
| H-mTBD-1 | 0.009 | 0.009 | - | |
| H-mTBD-2 | - | - | - | |

| | | | | |
|---|---|---|---|---|
| $^{-}$OAc | 0.157 | 0.151 | 0.002 | |
| K | 1.43E-05 | - | 1.47E-06 | |
| Na | 1.06E-05 | 1.55E-06 | 5.92E-06 | |
| Ca | 2.07E-05 | 5.64E-06 | 7.51E-06 | |
| Xylan | - | - | - | |
| Lactic acid | - | - | 6.7E-05 | |
| $\dot{m}$ = 0.480 kg/h | | | | |
| mTBD$^{+}$ | 0.337 | 0.328 | 0.006 | |
| TBD | 0.003 | 0.003 | - | |
| H-mTBD-1 | 0.007 | 0.007 | - | |
| H-mTBD-2 | - | - | - | |
| $^{-}$OAc | 0.132 | 0.129 | 0.002 | 0.002 |
| K | 1.19E-05 | 0.000 | 7.12E-06 | |
| Na | 8.86E-06 | 6.35E-07 | 1.27E-06 | |
| Ca | 1.74E-05 | 3.76E-06 | 1.153E-06 | |
| Xylan | - | - | - | |
| Lactic acid | - | - | - | |
| $\dot{m}$ = 0.378 kg/h | | | | |
| mTBD$^{+}$ | 0.265 | 0.247 | - | |
| TBD | 0.003 | 0.002 | - | |
| H-mTBD-1 | 0.006 | 0.008 | - | |
| H-mTBD-2 | - | - | - | |
| $^{-}$OAc | 0.104 | 0.097 | - | 0.023 |
| K | 9.44E-06 | - | - | |
| Na | 6.98E-06 | 4.60E-07 | - | |
| Ca | 1.37E-05 | 2.29E-06 | - | |
| Xylan | - | - | - | |
| Lactic acid | - | - | - | |

$\dot{m}_{feed}$: mass flowrate of feed, $\dot{m}_{distillate}$: mass flowrate of distillate, $\dot{m}_{residue}$: mass flowrate of residue, $\dot{m}_{losses}$: mass flowrate of losses; $\dot{m}_{feed} = \dot{m}_{distillate} + \dot{m}_{residue} + \dot{m}_{losses}$.
